# Factor XIII Deficiency in a Moroccan Infant: A Case Report of a Rare Bleeding Disorder

**DOI:** 10.7759/cureus.103326

**Published:** 2026-02-09

**Authors:** Khadija Mesbah, Yousra El Boussaadni, Abdallah Oulmaati

**Affiliations:** 1 Pediatrics, Centre Hospitalier Universitaire Mohammed VI de Tanger, Tangier, MAR

**Keywords:** bleeding disorder, factor xiii deficiency, factor xiii deficiency diagnosis, hereditary, postcircumcision bleeding

## Abstract

Factor XIII deficiency (FXIIID) is a rare autosomal recessive hereditary bleeding disorder caused by heterogeneous mutations, leading to potentially life-threatening hemorrhages and early fetal loss. FXIII plays a vital role not only in cross-linking fibrinogen to stabilize clot formation but also in wound healing, angiogenesis, and fetal viability. Diagnosing FXIIID is particularly challenging, as standard coagulation assays typically yield normal results, necessitating specific FXIII testing an additional barrier in resource-limited settings such as developing countries. Early diagnosis is crucial, especially due to the high risk of intracranial bleeding. We report the case of an infant who was admitted for postcirumcision bleeding and was ultimately diagnosed with congenital FXIII deficiency.

## Introduction

Factor XIII (FXIII) is more than a simple component of the coagulation cascade. Initially identified as fibrin-stabilizing factor (FSF), its primary known role was to cross-link fibrin and reinforce clot stability. However, recent studies have revealed broader biological functions, as FXIII is now recognized for its ability to cross-link a growing number of proteins not only in plasma but also within the vascular matrix, platelets, endothelial cells, and monocytes. This highlights the importance of intracellular FXIII in achieving hemostasis, thrombosis, and tissue repair [1].

Congenital FXIII deficiency, first described by Duckert et al. in 1960 [2], is a rare inherited bleeding disorder with an estimated prevalence of one in one to three million individuals [3]. It follows an autosomal recessive inheritance pattern and typically manifests when FXIII activity decreases below 1%, usually in homozygous or compound heterozygous individuals. The clinical presentation often begins in the neonatal period, commonly with prolonged umbilical cord bleeding. Compared with other congenital bleeding disorders, intracranial hemorrhage (ICH) is notably more common in FXIII deficiency patients, and other manifestations may include mucocutaneous bleeding, deep soft tissue hematomas, joint bleeding, and delayed wound healing. In many cases, bleeding can be delayed, occurring up to 36 hours after trauma or surgical procedures. Furthermore, FXIII deficiency is also associated with recurrent miscarriages due to impaired placental stability [4].

At the genetic level, FXIII deficiency is characterized by marked heterogeneity, with the most severe clinical phenotypes commonly associated with alterations of the FXIII A subunit, which contains the enzyme’s catalytic domain [5]. 

Through this case, we aim to emphasize the severity and diagnostic pitfalls of factor XIII deficiency and the need for heightened clinical awareness when routine coagulation tests are normal.

## Case presentation

A 24-month-old male infant, born via cesarean section to a 41-year-old mother (G5P5) with no history of consanguinity or familial bleeding disorders, presented with altered consciousness after sustaining a head trauma with an occipital point of impact.

**Figure 1:**
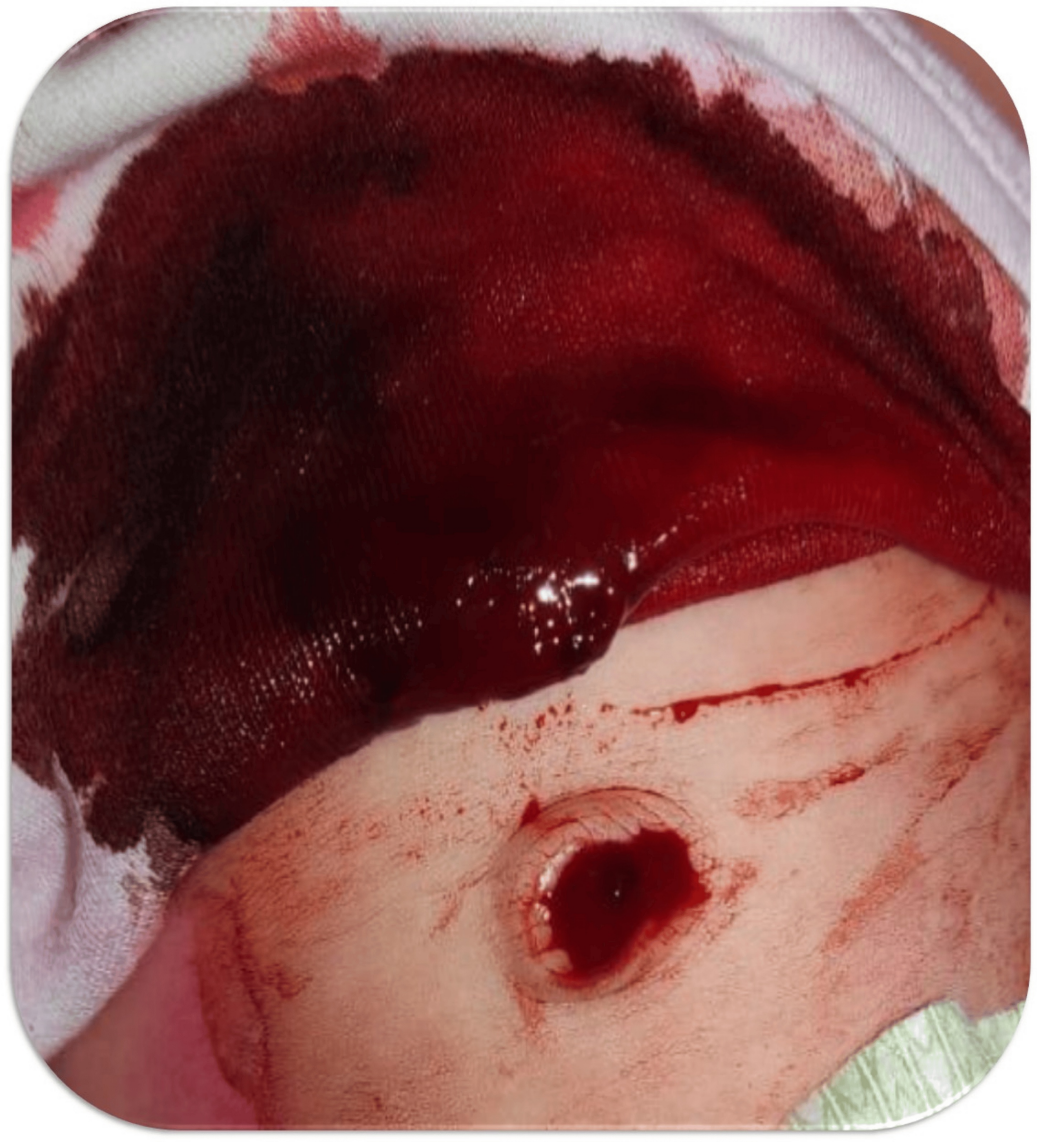
Umbilical hemorrhage secondary to umbilical cord separation

At 15 days of life, the baby had presented with significant umbilical bleeding requiring red blood cell transfusion (Figure 1). There was no history of hospitalization or antibiotic therapy at that point. The mother had been followed during pregnancy for hypothyroidism, managed with levothyroxine. She had initiated antituberculous therapy prior to pregnancy for cervical lymph node tuberculosis and continued treatment during the first two months of gestation. The infant had not received any anti-tuberculous treatment. The patient had been exclusively breastfed from birth. Vitamin K was administered orally at birth in accordance with standard prophylactic guidelines for the prevention of hemorrhagic disease of the newborn. No neonatal hepatic impairment was identified. The neonatal period was unremarkable. Initial coagulation parameters at presentation, including factor VIII, factor IX, von Willebrand factor, and FXIII levels, were normal. The bleeding resolved spontaneously.

At the age of four months, the infant had experienced an episode of rectal bleeding (rectorrhagia), which also resolved spontaneously without intervention. No further workup was performed at that time.

**Figure 2:**
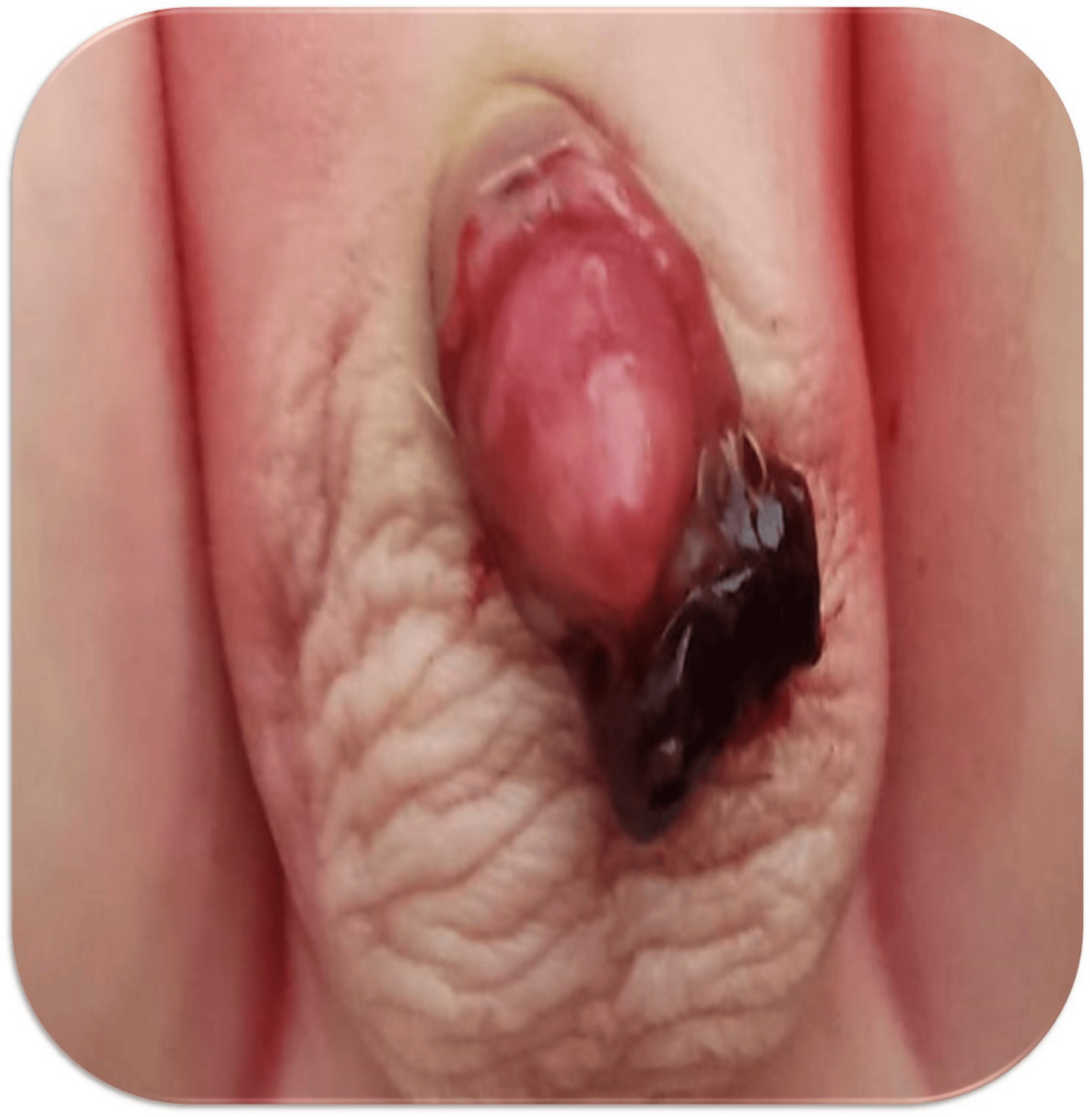
Post-circumcision hemorrhage showing active bleeding from the surgical site.

At 10 months of age, the patient underwent circumcision. Three days later, the patient presented with moderate postcircumcision bleeding, characterized by bright red blood (Figure 2). The prothrombin time (PT) and activated partial thromboplastin time (aPTT) were within normal limits. The FXIII level was 90%, interpreted as normal.

Repeat coagulation workup at 15 months of age revealed FXIII activity to be markedly decreased, with a measured value of 4% and a control of 6.7%, thereby confirming the diagnosis of congenital FXIII deficiency. Immunologic assays and von Willebrand factor activity were within the normal range, as was factor VII.

**Figure 3:**
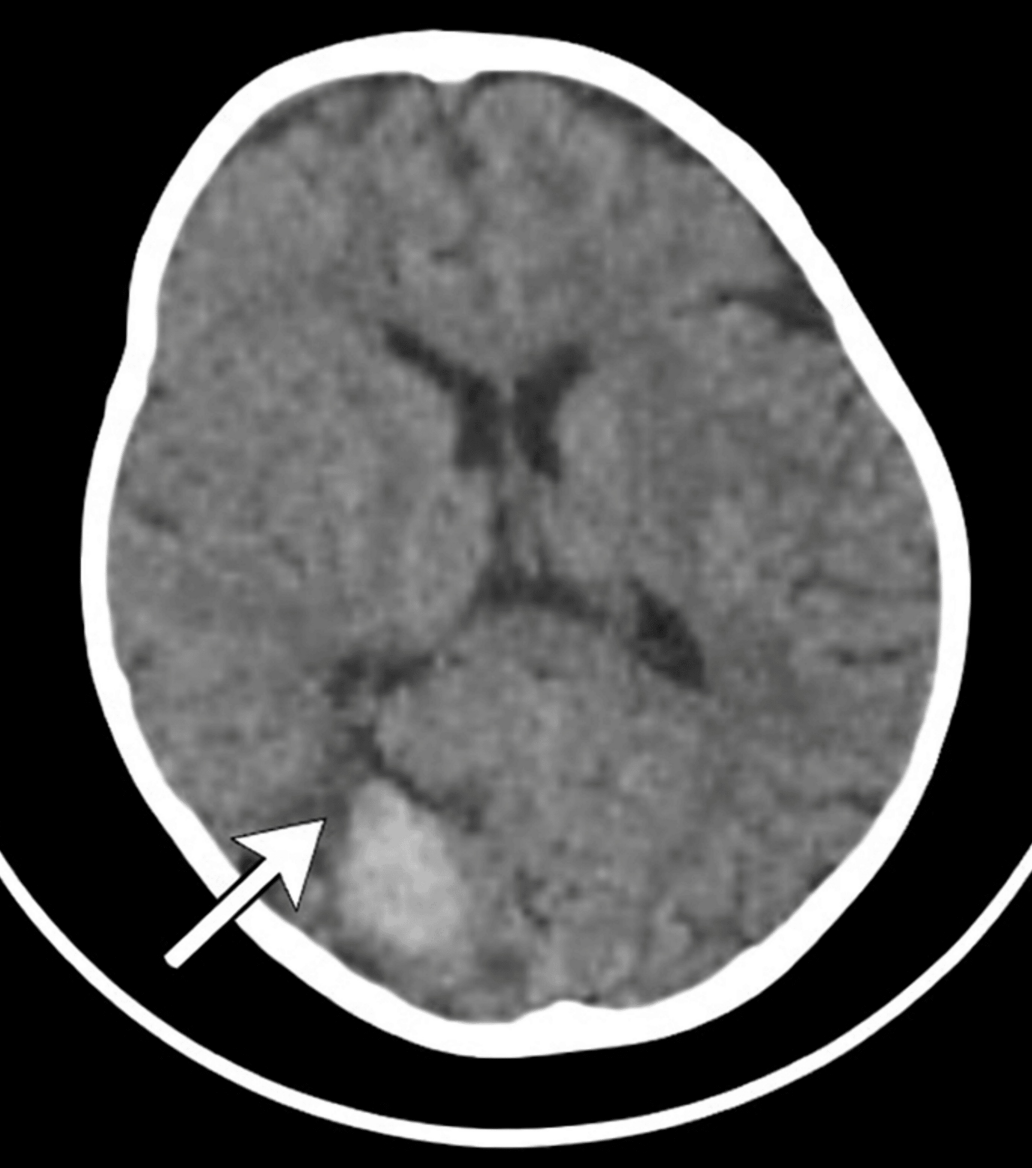
A brain CT scan revealed a right occipital intraparenchymal hematoma with a mass effect

At the current presentation with a head trauma at 24 months of age, a brain computed tomography (CT) scan was done, which revealed a right occipital intraparenchymal hematoma with a mass effect on the ipsilateral occipital horn of the lateral ventricle (Figure 3). An urgent request for fresh frozen plasma (FFP) was made, and the patient was treated with one unit of FFP every 12 hours for 10 days. A follow-up brain CT scan revealed regression of the hematoma.

A prophylactic regimen was initiated in the absence of specific replacement therapy and given the child’s vulnerable age and history of intraparenchymal hematoma, consisting of 20 cc/kg twice weekly, pending the availability of FXIII concentrate. The child is currently under regular follow-up in pediatric hematology at our institution.

## Discussion

Factor XIII deficiency is an uncommon bleeding disorder that frequently remains unrecognized because routine coagulation assays, including PT, aPTT, and bleeding time, are typically within normal ranges. The clinical presentation is highly variable, ranging from impaired umbilical cord healing in neonates to severe and potentially fatal hemorrhagic events such as intracranial bleeding. Once activated, factor XIII is essential for clot stabilization, as it reinforces the fibrin network through covalent cross-linking, thereby ensuring the formation of a durable and effective hemostatic plug [3].

Factor XIII deficiency may be congenital or acquired. The congenital form is inherited in an autosomal recessive pattern and most often results from mutations affecting the A subunit (FXIII-A), which harbors the enzymatic activity of the molecule, whereas acquired FXIII deficiency, which is more common, usually arises from conditions such as hyperconsumption, hemodilution, or impaired hepatic synthesis, and in rare cases from the development of autoantibodies against FXIII subunits. In inherited FXIII deficiency, abnormalities may involve either the A (FXIII-A) or B (FXIII-B) subunits; however, large series reported in the literature indicate that the majority of affected individuals predominantly exhibit FXIII-A deficiency [6].

Data from the International Registry on FXIII deficiency (n=104) show that the most frequent bleeding manifestations include subcutaneous bleeding (57%), delayed umbilical stump bleeding (56%), muscle hematoma (49%), postoperative bleeding (40%), hemarthrosis (36%), and intracerebral bleeding (34%) [7]. Similarly, the FranceCoag cohort (n=33; FXIII activity <10 IU/dL) reported life-threatening umbilical and/or intracranial hemorrhage in 62.1% of patients, with intracranial hemorrhage associated with neurological sequelae in some untreated cases [8]. In autoimmune-acquired FXIII deficiency, a large summary of reported cases (n=93) indicates a predominance in older adults, with approximately half of cases classified as idiopathic and the remainder associated with underlying conditions [9]. Overall, these series underscore that severe bleeding, particularly intracranial hemorrhage, is a major clinical hallmark of FXIII deficiency.

In our patient, the absence of a personal or family history of bleeding, the delayed onset of intracranial hemorrhage, and the initially normal neonatal FXIII activity initially raised consideration of a fluctuating measurement rather than true normal function. Neonatal FXIII assays may be unreliable or influenced by physiological variability, leading to falsely reassuring results. Repeat testing later demonstrated markedly reduced FXIII activity, confirming congenital FXIII deficiency. This case illustrates an important diagnostic pitfall: a normal neonatal FXIII level does not exclude congenital FXIII deficiency. Reliance on single-point testing may therefore be misleading and contribute to diagnostic delay.

Diagnosing FXIII deficiency remains a significant challenge, especially in resource-limited settings where specific FXIII assays are not routinely available. Consequently, FXIII deficiency is regarded as one of the most frequently overlooked rare coagulation disorders. Although FXIII was first identified in 1944 [10], its deficiency was not recognized clinically until 1960, largely because standard coagulation assays remain normal in affected patients. Early diagnostic approaches relied on the clot solubility test; however, this method is no longer recommended by current expert consensus. Accurate diagnosis now relies on functional activity assays and immunological quantification of FXIII levels [11,12]. Limited access to these tests contributes to underdiagnosis in many developing countries [13].

In such contexts, clinical suspicion becomes paramount. FXIII deficiency should be considered in patients presenting with unexplained severe or recurrent bleeding, particularly intracranial or postoperative hemorrhage, despite normal PT and aPTT, especially when bleeding is delayed or disproportionate to the initial trauma or surgical intervention. When FXIII testing is not immediately accessible, repeat testing, referral to specialized centers, or empiric management may be warranted, given the potentially life-threatening nature of the condition.

Therapeutically, although plasma-derived and recombinant FXIII concentrates have been available since the 1990s and 2010s [9,14], respectively, their use remains limited in many countries due to cost and accessibility [15]. In these settings, fresh frozen plasma remains the mainstay of treatment for acute bleeding. Given the long half-life of FXIII (approximately 9-14 days), prophylactic replacement therapy can effectively prevent recurrent bleeding in severe cases. The development of inhibitors against FXIII remains exceedingly rare, supporting favorable long-term management when the diagnosis is established [16].

## Conclusions

Congenital FXIII deficiency, a perfect example of “hidden bleeding”, although rare, poses a significant risk of severe and sometimes life-threatening bleeding, particularly in the neonatal period and after surgical procedures. Despite these risks, the overall prognosis is favorable owing to the efficacy of available treatments such as FFP, cryoprecipitate, or plasma-derived FXIII concentrates. This case underscores the importance of maintaining a high index of suspicion for FXIII deficiency in infants presenting with unexplained or delayed bleeding, especially when routine coagulation tests are normal. 
